# Spike train distance metric reveals plasticity in discrimination of salient calls by putative excitatory cells of the auditory cortex

**DOI:** 10.1186/1471-2202-13-S1-P97

**Published:** 2012-07-16

**Authors:** Charles L Zhao, Frank Lin, Robert C Liu

**Affiliations:** 1 Biomedical Engineering Graduate Program, Coulter Department of Biomedical Engineering at Georgia Institute of Technology and Emory University, Atlanta, GA 30332, USA; 2 Interdisciplinary Bioengineering Graduate Program, Coulter Department of Biomedical Engineering at Georgia Institute of Technology and Emory University, Atlanta, GA 30332, USA; 3 Department of Biology, Emory University, Atlanta, GA 30322, USA

## 

Despite significant progress in recent years in characterizing the response properties of neurons in the auditory cortex, the exact role that auditory cortical activity plays in sound processing remains highly debated. One hypothesized function is to improve the detection, discrimination or categorization of sounds that are behaviorally salient. We explore this possibility in a mouse model using species-specific ultrasonic calls. Single unit cortical recordings were taken in awake, head-restrained virgin and mother mice during playback of a variety of conspecific pup and adult calls. Previously [1], we had uncovered plasticity in the inhibition evoked by pup calls as they gained salience, observing changes from virgin to mother mice, the latter which recognize the importance of pup calls. Qualitatively, recorded putative excitatory neurons (based on spike waveform) also appeared different between animal groups in the call-evoked spike rasters. Here, we apply a novel normalization of spike train distances to quantify these differences and demonstrate plasticity in the functional call processing from evoked excitation.

We used the Van Rossum and Victora-Purpura distance metrics to quantify differences between the “collapsed” spike trains (i.e. pooled across trials) evoked by different calls. We noticed that the magnitude of these distances were often dominated by overall evoked spike rate, which complicated comparisons across units with different degrees of call responsiveness. Hence, to obtain a clearer picture of changes between animal groups regardless of overall responsiveness (which was variable), we created for the distance between any pair of calls, a normalized spike distance between their “collapsed” spike trains. Specifically, the usual distance was divided by the average of the distance between spontaneous spike trains and those evoked by each call, thus quantifying average responsiveness to calls in terms of evoked spike train distances from silence.

Our results continue to support the idea that the contrast in neural population encoding for these calls across core auditory cortical fields has improved. As in the case of evoked inhibition, the most pronounced changes arise in the lateral-band areas not normally tuned for the ultrasonic frequencies of the calls studied (See Fig. 1). Taken together, this leads us to hypothesize that acquiring recognition of a class of vocalizations is correlated with refinement in the core auditory cortical representation of these sounds.

**Figure 1:**
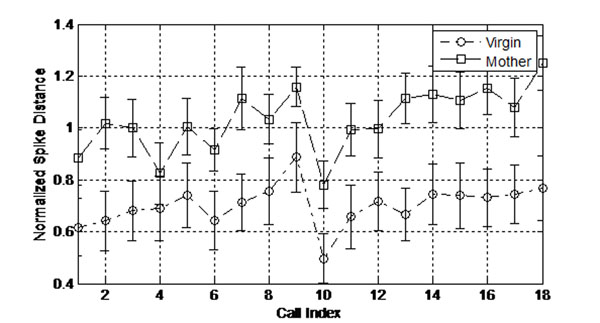
Normalized Victor-Purpura distance between adult and pup calls of equivalent frequency, duration, amplitude, and initial frequency sweep. For each group of animals, these values were averaged over all single units from the Primary and Anterior Auditory Fields, considered the lateral band for ultrasonic calls. Call Index refers to a comparison between 1 of 18 pup calls to its matched adult call.

## References

[B1] Galindo-Leon EE Lin FG Liu RC Inhibitory plasticity in a lateral band improves cortical detection of natural vocalizations Neuron 2009 62 5 705 16 10.1016/j.neuron.2009.05.001 19524529 PMC2709999

